# Enhanced Accuracy of Airborne Volcanic Ash Detection Using the GEOKOMPSAT-2A Satellite

**DOI:** 10.3390/s21041359

**Published:** 2021-02-15

**Authors:** Soi Ahn, Joon-Bum Jee, Kyu-Tae Lee, Hyun-Jong Oh

**Affiliations:** 1 National Meteorological Satellite Center (NMSC), Korea Meteorological Administration (KMA), Jincheon-gun 27803, Korea; jjahn@korea.kr; 2 Research Center for Atmospheric Environment, Hankuk University of Foreign Studies, Yongin 17035, Korea; rokmcjjb717@gmail.com; 3 Department of Atmospheric & Environmental Sciences, Gangneung-Wonju National University, Gangneung 25457, Korea; ktlee@gwnu.ac.kr

**Keywords:** remote sensing, volcanic ash, GEOKOMPSAT-2A, AMI, volcanoes, earth observation

## Abstract

In this study, a technique facilitating the enhanced detection of airborne volcanic ash (VA) has been developed, which is based on the use of visible (VIS), near-infrared (NIR), and infrared (IR) bands by meteorological satellite systems. Channels with NIR and IR bands centered at ~3.8, 7.3, 8.7, 10.5, and 12.3 μm are utilized, which enhances the accuracy of VA detection. The technique is based on two-band brightness temperature differences (BTDs), two-band brightness temperature ratios (BTRs), and background image BTDs. The physical effects of the observed BTDs and BTRs, which can be used to distinguish VA from meteorological clouds based on absorption differences, depend on the channel and time of day. The Advanced Meteorological Imager onboard the GEOKOMPSAT-2A (GK-2A) satellite has several advantages, including the day- and nighttime detection of land and ocean. Based on the GK-2A data on several volcanic eruptions, multispectral data are more sensitive to volcanic clouds than ice and water clouds, ensuring the detection of VA. They can also be used as an input to provide detailed information about volcanoes, such as the height of the VA layer and VA mass. The GK-2A was optimized, and an improved ash algorithm was established by focusing on the volcanic eruptions that occurred in 2020. In particular, the 3.8 μm band was utilized, the threshold was changed by division between day and night, and efforts were made to reduce the effects of clouds and the discontinuity between land and ocean. The GK-2A imagery was used to study volcanic clouds related to the eruptions of Taal, Philippines, on 12 January and Nishinoshima, Japan, from 30 July–2 August to demonstrate the applicability of this product during volcanic events. The improved VA product of GK-2A provides vital information, helping forecasters to locate VA as well as guidance for the aviation industry in preventing dangerous and expensive interactions between aircrafts and VA.

## 1. Introduction

During volcanic eruptions, excessive amounts of volcanic ash (VA), aerosols, and gases are released into the atmosphere, presenting both human health and aviation issues. The primary concern is the risk of an engine shutdown due to the melting of VA particles within the engine of an aircraft. The VA particles can also generate extensive damage to the frame of an aircraft due to scraping [1]. They pose severe hazards to high-altitude jet aircrafts along major air routes adjacent to active volcanoes. Ash clouds may persist for many hours or days, leading to flight route diversions in regions thousands of kilometers from their source. Timely and accurate information about the location and concentration of VA helps pilots to avoid this type of hazard [2]. However, in satellite imagery, volcanic clouds are often obscured by meteorological clouds or are too small to be detected [3]. In addition, satellite images are affected by tropopause-overshooting clouds, water vapor, weak ash plumes, and instrument noise [4,5].

Satellite remote sensing technology has been developed to predict volcanic eruptions. Oppenheimer et al. [6] discussed remote sensing techniques that have been used in volcanic monitoring since the early 1980s. Both visible (VIS; daytime) and infrared (IR; day and night) bands are used by geostationary satellites at high spatial resolution. However, volcanic and meteorological clouds cannot be distinguished based on these bands, and thus, the importance of IR channels has increased. Prata et al. [7] utilized the observed brightness temperature difference (BTD) between the “split window” IR band centered at a wavelength of ~12.0 μm and the IR window at 11 μm, that is, reverse absorption (RA). Based on this method, VA and meteorological clouds can be distinguished. The RA method uses Geostationary Operational Environmental Satellites (GOES) with sensors in the IR range to monitor eruptions [8,9,10]. However, this method is limited to bright surfaces during the daytime [4] and the atmosphere above water during the night, and thus, it is difficult to detect smaller eruptions [10]. Therefore, modified methods have been developed, such as the two-band brightness temperature ratio (BTR) [11], three-band VA product (TVAP) based on the wavelengths 3.7, 10.8, and 12.0 μm [12], and hybrid algorithms based on RA and TVAP [13]. In addition, there have been increased attempts to use various channels such as the four channels based on IR brightness temperature (BT) and visible reflectance [14]. 

In this study, we used thermal GEOKOMPSAT-2A (GK-2A) data to demonstrate that it is possible to detect and discriminate volcanic clouds. The GK-2A shortwave infrared (SWIR) band 7 (3.8 μm) of the Advanced Meteorological Imager (AMI) is a window characterized by little or no moisture absorption and a very high sensitivity to heat sources (such as fires or volcanoes). The 3.8 μm band also has a strong daytime solar reflectance component, which can interfere with the emitted thermal energy of several types of clouds or surface features [15]. The SWIR and longwave IR (LWIR) BTD has been used to distinguish low-level fog and stratus clouds from dense cirrus, high-level ice clouds, and background, both during day and nighttime [16]. Water clouds in the SWIR region can also be distinguished from ice clouds based on their reflectance. The reflectance of water clouds in the SWIR region is inversely proportional to the effective radius of the cloud particles in the range of 5–20 μm [17,18], and is larger than the reflectance of ice clouds composed of particles with similar sizes. Clouds comprising small water droplets are more reflective than those consisting of either large water droplets or ice particles. Therefore, lower-level eruption clouds, which are mainly composed of ash and water droplets, will be significantly enhanced by the utilization of 3.8 μm of GK-2A [12]. 

Recently, volcanic eruptions occurred in Japan and the Philippines region close to Korea, and thus, it is important to monitor volcanoes [19,20,21,22]. In this study, a more advanced algorithm is established to monitor and analyze the volcanic eruptions that occurred in 2020. Based on the application of the new algorithm to volcanic eruptions, volcanic clouds can be detected using the GK-2A multispectral data.

This paper is organized as follows. An overview of ash retrieval schemes is presented in Section 2. The GK-2A satellite instruments used for the measurements are described in Section 3, and test cases are introduced. The findings are summarized in Section 4. 

## 2. Data and Methods 

### 2.1. Data

#### 2.1.1. GEOKOMPSAT-2A (GK-2A)

The Korea Meteorological Administration (KMA) initiated the continuation of the Korean Communication, Ocean, Meteorological Satellite (COMS) mission using the follow-on geostationary meteorological satellite GK-2A. The GK-2A AMI carries out measurements with high spatiotemporal resolution (0.5~2 km) every 2 min over the Korean Peninsula and every 10 min in the full-disk. After its successful launch on 5 December 2018 and an in-orbit test that lasted ~8 months, GK-2A, including a 16-channel AMI, was commissioned on 25 July 2019 (Table 1). The GK-2A is similar to the Japanese Himawari-8 and the United States GOES-16 satellites, which were launched in 2014 and 2016, respectively. The AMI has a VIS channel with a resolution of 1 km (0.43, 0.50, 0.63, and 0.85 μm), a near infrared channel (1.38 and 1.60 μm), and an IR channel with a resolution of 2 km (3.8, 6.3, 6.9, 7.3, 8.7, 9.6, 10.5, 11.2, 12.3, and 13.3 μm). It is used for diverse observations to improve the monitoring of meteorological phenomena and facilitates faster and continuous monitoring of the atmospheric environment. 

#### 2.1.2. Validation Datasets

Based on the assumption that sulfur dioxide is generated during a volcanic eruption, sulfur dioxide data from other satellites were used to verify the ash output. The Support to Aviation Control Service (SACS) hosted by the Royal Belgian Institute for Space Aeronomy (BIRA-IASB) is a project initiated by the European Space Agency (ESA), which aims at supporting the VA Advisory Center (VAAC). To validate the VA Product (VAP) of GK-2A, we used the SO_2_ and VA data derived from satellite UV (Ultraviolet)—Visible including OMI (Ozone Monitoring Instrument), GOME 2A&B (Global Ozone Monitoring Experiment), OMPS (Ozone Mapping and Profiler Suite), TROPOMI (TROPOspheric Monitoring Instrument) and IR including AIRS (Atmospheric Infrared Sounder), IASI-A/B (Infrared Atmospheric Sounding Interferometer) instruments (https://sacs.aeronomie.be/) (accessed on 10 February 2021). We also used Cloud-Aerosol Lidar and Infrared Pathfinder Satellite (CALIPSO) data, including observations of clouds and aerosols and IR imagery of high cirrus clouds in this region. These data provide information about the vertical distribution of VA, that is, the altitude of VA [23,24,25,26,27,28,29].

#### 2.1.3. Volcanic Ash 2020

In this study, we focused on volcanic events that occurred in 2020 in East Asia because the GK-2A satellite data are available for this year and can be optimized based on specific cases. On 12 January 2020, a large volcanic eruption occurred in Taal in the Philippines, causing significant damage and leading to the evacuation of thousands of people. Several small explosions occurred in Indonesia after the main eruption, causing anxiety amongst people. In addition, long-term volcanic eruptions that occurred from 30 July to early August 2020 on Japan’s Nishinoshima island affected the atmosphere in East Asia. Volcanic eruptions occur in this region to this day. 

### 2.2. Method

#### 2.2.1. Volcanic Ash Algorithm

**Figure 1:**
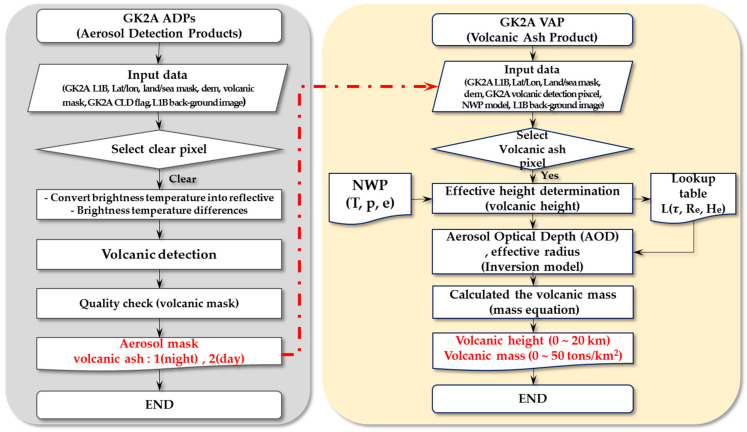
Flowchart of the volcanic ash product algorithm used by GK-2A/AMI.

During the development of the automated algorithm presented in this paper, our goal was to minimize the limitations by utilizing a multi-channel technique based on additional spectral information. The VAP algorithm flowchart is shown in Figure 1. The VAP algorithm includes the preparation of input data, a data quality check, effective height determination for the selection of a suitable lookup table (LUT), the retrieval of VA with the inversion model, the evaluation of the retrieval quality, and output (VA mass, VAM) production.

##### Aerosol Optical Depth Lookup Table

It is critical to calculate an LUT using a well-defined VA aerosol model to acquire an accurate aerosol optical depth (AOD) because the measured top of atmosphere (TOA) radiance is significantly affected by the concentration and microphysical properties of VA aerosols. Table 2 shows the node points for the calculation of the TOA radiance using the Santa Barbara discrete ordinate radiative transfer (SBDART) code of the libRadtran software package (http://libradtran.org) (accessed on 10 February 2021) [30,31]. To establish 10 VA aerosol models from normalized VA size distributions (with 10 effective radius) from the WCP (World Climate research Programme)-112 [32] measurement data, the LUTs were calculated using the radiative transfer model. Because the measured TOA radiance consists of scattering due to surface emissivity and the atmosphere including aerosols, the TOA radiance was calculated and an LUT was constructed as a function of the VA AOD, optical properties of the VA aerosols [28], wavelength, extinction coefficient, and single-scattering albedo [33]. 

##### Background Composite Image

The background image was underestimated and aerosol scattering was not completely removed from the measured TOA reflectance, resulting in a less accurate retrieved AOD. The minimum of the 30-day reflectance values was regarded as the surface reflectance based on the assumption that each pixel contains at least one clear condition without clouds or aerosols. However, the reflectance signal decreases close to the critical reflectance due to changes in the aerosol loading. It decreases with increasing AOD and the surface reflectance is larger than the critical reflectance [33,34]. In addition, the calculation method of the maximum of the 30-day BT values in IR was changed to a 30-day average composite in order to collectively represent the change in the surface temperature and the season. Therefore, the accurate calculation of the background field can lead to an increase in the accuracy of the VA detection. The background field of the VIS and IR channels 30 days before the observation was calculated and applied. On that day, the aerosol fluctuation was the smallest, which reduces the limitations due to the change in the season. 

##### Cloud Masking and Quality Assurance

Cloud screening tests can be used to distinguish cloud and low-aerosol pixels from those related to moderate and heavy aerosol conditions. Using the observed spectral VIS reflectance and IR BT, the algorithm searches for spectral signatures due to clouds. To prevent the removal of the aerosol signal by clouds, a cloud threshold that can only be used for aerosol detection was determined and screened; it expands the range of the boundary value [35]. 

Based on the use of the VIS–IR channels (Equations (1)–(3)),

(1) ρ0.4<0.4, ρ0.6<0.4, ρ1.6<0.4

(2) $$\rho 1.3<0.035,\;BT_{11.2}<270\;K$$

(3) $$BT_{6.9}<220.0\;K,\;BT_{10.5}<265.0\;K,\;BT_{13.3}<224.0\;K$$

where *ρ*_0.4_, *ρ*_0.6_, *ρ*_1.3_, and *ρ*_1.6_ represent the reflectance at 0.4, 0.6, 1.3, and 1.6 μm, respectively (*ρ*; *wavelength*), and *BT*_6.9_, *BT*_10.5_, *BT*_11.2_, and *BT*_13.3_ represent the *BT* at 6.9, 10.5, 11.2, and 13.3 μm, respectively.

In the ocean (Equations (4) and (5)),

(4) $$\left(\mathrm{Max}BT_{10.5}-BT_{10.5}\right)-3.0\times 7.0\times \left(\frac{dem}{1000}\right)\times stdev>3.2$$

(5) $$\mathrm{ABS}\left((BT_{10.5}-BT_{12.3}\right)-\left(\mathrm{Max}BT_{10.5}-MaxBT_{12.3}\right))>0.7$$

On land (Equations (6) and (7)),

(6) $$\left(\mathrm{Max}BT_{10.5}-BT_{10.5}\right)-3.0\times 7.0\times \left(\frac{dem}{1000}\right)\times stdev>4.1$$

(7) $$\mathrm{ABS}\left((BT_{10.5}-BT_{12.3}\right)-\left(\mathrm{Max}BT_{10.5}-\mathrm{Max}BT_{12.3}\right))>1.0$$

where *BT*_10.5_ and *BT*_12.3_ represent the *BT* at 10.5 and 12.3 μm, respectively, Max *BT*_10.5_ and Max *BT*_12.3_ represent the Max *BT* at 10.5 and 12.3 μm in the background 30-day image before detection (the maximum *BT* in 30-day background image), and *dem* is the geological altitude (m) based on the digital elevation model (above sea level). ABS means absolute value.

##### Volcanic Ash Detection Using a Multi-Channel Threshold Test

**Figure 2:**
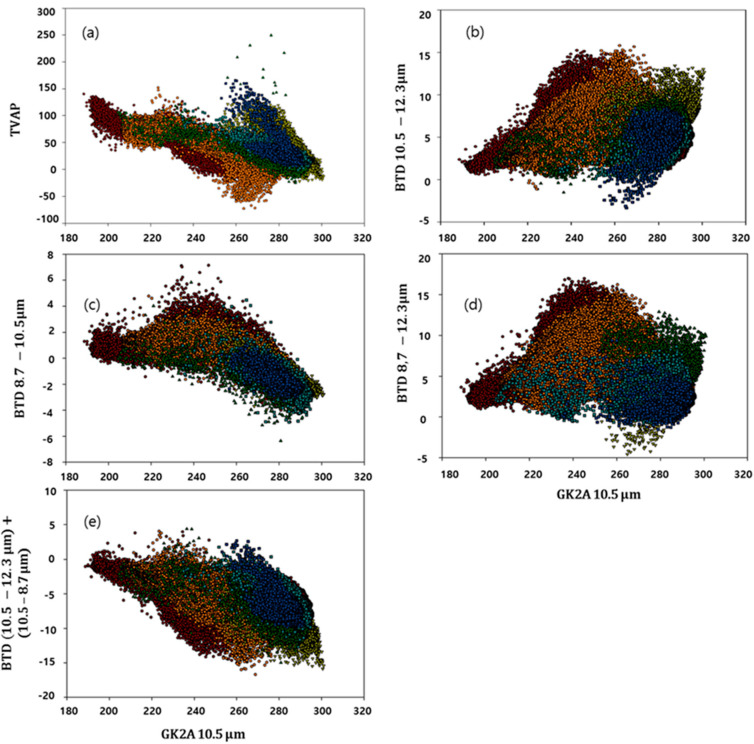
Brightness temperature (BT) versus BT difference (BTD) at 10.5 μm. (**a**) Three-band VA product (TVAP), (**b**) 10.5–12.3 μm (**c**) 8.7–10.5 μm (**d**) 8.7–12.3 μm (**e**) Ash RGB threshold of GK-2A/AMI for the measurements of the Taal (red, January 12), Merapi (yellow, March 2), Merapi (green, March 27), Krakatau (sky blue, April 10), Semeru (orange, May 16), and Nishinoshima (blue, July 31) eruptions.

Because the GK-2A VAP algorithm described in this section uses data from multiple spectral channels, we apply to the algorithm as the improved GK-2A VAP algorithm. To determine the characteristics of the volcano and optimize the threshold, the *BT* of each channel of the GK-2A was analyzed a spot within a radius of 0.5° from the point at which the volcano occurred, focusing on the 2020 eruption (Table 3). Six representative volcanic eruptions that occurred in 2020 were selected and analyzed using the GK-2A 10.5 μm band on the *x*-axis and BTD and BTR values for the detection on the *y*-axis. In this study, we focused on identifying a common boundary value based on the clean channel (10.5 μm). Based on the analysis of six cases, the following boundary values were determined (Figure 2a, Equations (8) and (9)):

(8) $$\mathrm{TVAP}=C+m_{1}\left(12.3\;\mu m-10.5\;\mu m\right)+m_{2}\left(3.8\;\mu m-10.5\;\mu m\right)$$

(9) TVAP(Day)<75 K, TVAP(Night)<70 K

where C is a constant (60) and m_1_ and m_2_ are scaling factors (10 and 3, respectively) obtained from principal component analysis (PCA).

Static thresholds are generally used for the BTD 10.5–12.3 tests, which are the most useful for detecting volcanic clouds that reside in the upper troposphere or volcanic clouds in a dry atmosphere, which is typical for high latitudes [36]. However, in contrast to the common implementation of the RA technique, constraints from additional spectral channels are used for the tests to reduce false alarms [35]. These constraints, which are selected based on the spectral properties, prevent the false identification of non-volcanic aerosols and very high cloud tops as VA. In this study, RA was used as a threshold, especially for volcanoes with strong eruptions such as the Taal volcano, leading to a significant difference with respect to the results. In addition, this method is the most sensitive to fine ash, which is important in the case of volcanoes containing large concentrations of sulfur dioxide such as the Nishinoshima volcano. Figure 2b shows the volcanic plume 1 h after the eruption of the six volcanoes. The results for the split-window channels are shown in Figure 2b, in which the BTD 10.5–12.3 is plotted against BT10.5. Although the negative difference is small, a characteristic inverted “U” shape can be observed. This is evidence for partial or semi-transparent clouds [7], indicating that the six volcanic clouds formed by mixing with other clouds. However, at a BT [10.5] value of ~270–280 K, a large positive difference is expected for water-ice clouds. Therefore, by only identifying volcanic clouds that are mixed with other clouds, when the RA method was used, the false detections significantly increased, and thus other channels were utilized for the threshold test (Equations (10) and (11)):

(10) $$BT_{10.5}>190.0\;K$$

(11) $$BT_{10.5}-BT_{12.3}<-2.0\;K$$

The BTD 8.7–10.5 and BTD 8.7–12.3 tests were based on thresholds differentiating between the absorption of ice clouds and aerosols. In particular, the BTD 8.7–12.3 test can be used to monitor stratospheric volcanic aerosols based on observations at 8.7 and 12.3 μm [37]. The volcanic ash transmission at 8.7 and 12.3 μm is almost similar, but the single-scattering albedo is less than 0.1 at 8.7 μm, and thus the optical depth is dominated by absorption [38]. The wavelength of 8.7 μm is potentially the most useful because it absorbs more water vapor than other gases. Thus, the 8.7 μm band can be used to retrieve the SO_2_ in the lower troposphere and in a relatively transparent region. Therefore, a threshold sensitive to the presence of H_2_SO_2_/H_2_O aerosol was used (Figure 2c,d, Equations (12) and (13)):

(12) $$BT_{8.7}-BT_{10.5}<0.0\;K$$

(13) $$BT_{8.7}-BT_{12.3}<5.0\;K$$

The combined BTD 10.5–12.3 and BTD 8.7–10.5 test is a three-channel BTD, which was developed using the Ash RGB (Red-Green-Blue) imager (Equation (14)). The threshold of 0.0 K was determined based on comparisons with time-varying loops of the Ash RGB images of the six eruptions described above (Figure 2e). In a previous study, the thresholds were set to +1.5 K; however, smaller thresholds were used in this study [39]. These differences should affect the 30-day background image since its characteristics are different for each season—it was calculated as an average field to minimize the impact.

(14) $$\left(BT_{8.7}-BT_{10.5}\right)+\left(BT_{12.3}-BBT_{10.5}\right)<0.0\;K$$

The BBTD–BTD (background) test was similar to the combined BTD 12.3–10.5 and BTD 8.7–10.5 test. However, it includes a water vapor correction [39,40,41,42]. For this correction, 30-day background image data were used to calculate the clear-sky BTs at 8.7, 10.5, and 12.3 μm (Equations (15)–(18)).

(15) $$3.0\;K<\left(BT_{12.3}-BT_{10.5}\right)-\left(BBT_{12.3}-BBT_{10.5}\right)<4.0\;K\;\left(\mathrm{Day}\right)$$

(16) $$2.0\;K<\left(BT_{12.3}-BT_{10.5}\right)-\left(BBT_{12.3}-BBT_{10.5}\right)<3.0\;K\;\left(\mathrm{Night}\right)$$

(17) $$0.0\;K<\left(BT_{8.7}-BT_{10.5}\right)-\left(BBT_{8.7}-BBT_{10.5}\right)<2.0\;K\;\left(\mathrm{Day}\right)$$

(18) $$-1.3\;K<\left(BT_{8.7}-BT_{10.5}\right)-\left(BBT_{8.7}-BBT_{10.5}\right)<0.0\;K\;\left(\mathrm{Night}\right)$$

where *BT* is the brightness temperature and *BBT* is the background brightness temperature.

**Figure 3:**
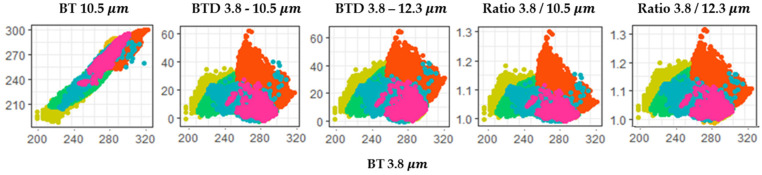
Scatter plot of the brightness temperature (BT) at 3.8 and 10.5 μm, BT difference (BTD) between 3.8–10.5 and 3.8–12.3 μm, and ratio of the BT at 3.8/10.5 and 3.8/12.3 μm for six volcanoes in 2020 (Taal (yellow), Merapi (orange), Merapi_v2 (orange red), Krakatau (sky blue), Semeru (green), and Nishinoshima (deep pink)).

Because of the complex radiative processes at the wavelength of 3.8 μm during the day, a TVAP threshold that can be used to distinguish VA from meteorological clouds has not been established. However, most of the selected eruptions occurred at night. Therefore, by utilizing the wavelength of 3.8 μm and a new threshold, the detection accuracy could be improved. The use of the 3.8 μm band for VA detection based on differential absorption and transmittance at night leads to smaller BTDs. However, they are relatively larger than those of cloud and surface features. Figure 3 shows the scatter plot of BTD 3.8–10.5, BTD 3.8–12.3, Ratio 3.8/10.4, and Ratio 3.8/12.3 as a function of the wavelength (3.8 and 10.5 μm, respectively). The relationship between various variables at ~3.8 μm was determined and a common threshold was identified based on the scatter plots. This is to find the common threshold of the Ash cases used in this study, and a correlation of 12.3 μm was newly applied in case of the utilization of 3.8 μm. Ash clouds have a lower BT at 10.5 and 12.3 μm (LWIR) than meteorological clouds, and the reflectance from all surfaces has a higher BT at 3.8 μm (SWIR). Therefore, threshold was added to detect VA using the property (Equation (19)). A low-intensity volcanic eruption can be distinguished from low-level fog and water cumulus; when it rises high, similar to overshooting clouds, it can be discriminated from stratus clouds, dense cirrus, and high clouds. In addition, the reflectance of the ash cloud is higher than that in the LWIR region. The SWIR/LWIR ratio can be used to increase the accuracy of the boundary test (Equations (19) and (20)):

(19) $$1.0\;K<BT_{3.8}-BT_{10.5}<20.0\;K,\;1.0\;K<BT_{3.8}-BT_{12.3}<30.0\;K$$

(20) $$\mathrm{Ratio}\left[\frac{3.8\;\mu m}{10.5\;\mu m}\right]>1.0,\;\mathrm{Ratio}\left[\frac{3.8\;\mu m}{12.3\;\mu m}\right]>1.0$$

The biconical reflectance of the VA samples is 20%–50% higher at 3.8 μm than in the IR region [43]. To emphasize the effect of the reflectivity, the 3.8 μm band was converted to reflectivity. Visible reflectance ratios, such as (ρ_3.8/_ρ_0.6_), during the daytime were utilized for the conversion [44,45,46].

The *BT* was converted into reflectivity as follows (Equation (21)):

(21) $$\mathrm{Ratio}\left[3.8\;\mu m\right]=L\left[3.8\;\mu m\right]-\frac{BT_{10.5}}{L_{0}*u\;-\;BT_{10.5}}$$

where *L* [3.8] is the radiance observed at 3.8 μm; *BT* 10.4 is the Planck radiation formula at 3.8 μm, which is calculated using the BT observed at 10.4 μm; Lo is the solar constant at 3.8 μm; and u is the cosine of the solar zenith angle. The information based on R [0.66] and R [3.8] can be combined into Ratio 3.8/0.66 to provide additional quantitative information about the presence of VA. Ratio 3.8/0.66 of VA-dominated clouds is greater than that of water- or ice-dominated clouds. Therefore, the algorithm uses a threshold test to increase the detection accuracy during the daytime (Equation (22)):

(22) $$\mathrm{Ratio}\left[\frac{3.8\;\mu m}{0.66\;\mu m}\right]>0.1$$

##### Determination of the Effective Volcanic Ash Cloud Height

The retrieved *T_eff_* was used to approximate the ash cloud height to find the nearest temperature point. The calculation method of this study was applied in the same way as the GOES-R Volcanic ash height method, and the linear interpolation weights and points were determined by locating *T_eff_* within the NWP (Numerical Weather Prediction) temperature profile, which searched from high to low vertical levels. The vertical NWP profiles used for the ash retrievals reflected the levels between the surface and model tropopause height which can most specify that temperature. The weights and points were then used to determine the ash cloud height based on interpolation (Equation (23)):

(23) $$Z_{ash}\left(\mathrm{height}\right)=Z_{1}+\frac{T_{eff}-T_{1}}{T_{2}-T_{1}}\left(Z_{2}-Z_{1}\right)$$

where *Z_ash_* is the ash cloud height; *T*_1_ and *T*_2_ are the temperatures within the profile that bound *T*_eff_, with *T*_1_ being the temperature at the highest bounding level (e.g., furthest from the ground); and *Z*_1_ and *Z*_2_ are the heights of the bounding temperatures corresponding to *T*_1_ and *T*_2_, respectively [47].

##### Calculation of the Volcanic Ash Mass Loading

**Figure 4:**
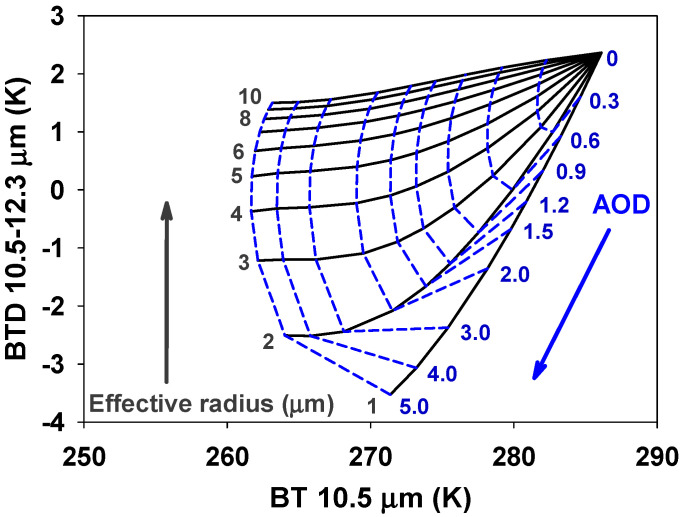
Example of a lookup table for the determination of the volcanic ash optical depth and effective radius (when altitude 2 km, solar zenith angle 30°, satellite angle 30°, and relative azimuth angle 80°).

The computation of the ash mass is based on the methodology reported by Zhang et al. [48]. The ash mass was computed from the cloud emissivity retrieved at 11 μm. Ratio 10.5/12.3 was used to obtain the AOD and effective radius (μm; Figure 4). The retrieval was based on searching for the closest value in the LUT that was calculated from the satellite-observed BTs.

To be chosen as closest to the observation, a set of BTs within the LUT must have values similar to the observations in each channel. The differences between the calculated and observed BTs must be similar to the differences between the observed BTs, as indicated by the root-mean-square deviation (RMSD) (Equation (24)):

(24) $$\mathrm{RMSD}=\frac{1}{N}\sqrt{{\frac{\left(BT_{calc}-BT_{obs}\right)}{BT_{obs}}}^{2}}+\frac{1}{N}\sqrt{{\frac{\left(BTD_{calc}-BTD_{obs}\right)}{BTD_{obs}}}^{2}}$$

where *BT_calc_* is the *BT* calculated with the radiative transfer model, *BT_obs_* is the satellite-observed *BT*, *BTD_calc_* is the *BTD* calculated with the radiative transfer model, and *BTD_obs_* is the satellite-observed *BTD*.

The total number of particles per unit area was calculated from the AOD and extinction cross sections at 11 μm (Equation (25)):

(25) $$N_{0}=\frac{\tau \left(11\;\mu m\right)}{\sigma \left(11\;\mu m\right)}$$

where τ is the AOD and σ is the effective radius.

Finally, the ash mass was computed as follows (Equation (26)):

(26) $$Ash\;Mass=\left(1\times 10^{6}\right)[\frac{4}{3}\pi \rho_{ash}\int_{r1}^{r2}r^{3}n\left(r\right)dr]\left(1\times 10^{-6}\right)$$

where the Ash Mass is the mass loading in t/km^2^; *p_ash_* is the density of the ash, which is 2.6 g/cm^3^ [49]; r is the particle radius, which is expressed in μm; n(r) is the number of particles per μm^2^; and the factor 1 × 10^6^ is used to convert the unit in t/km^2^. The integral is numerically evaluated using the rectangle rule quadrature.

## 3. Results

We studied six volcanoes that erupted in 2020 and characterized the atmosphere after each eruption.

### 3.1. Algorithm Performance

#### 3.1.1. Case 1: Taal Volcano Eruption (12 January 2020)

**Figure 5:**
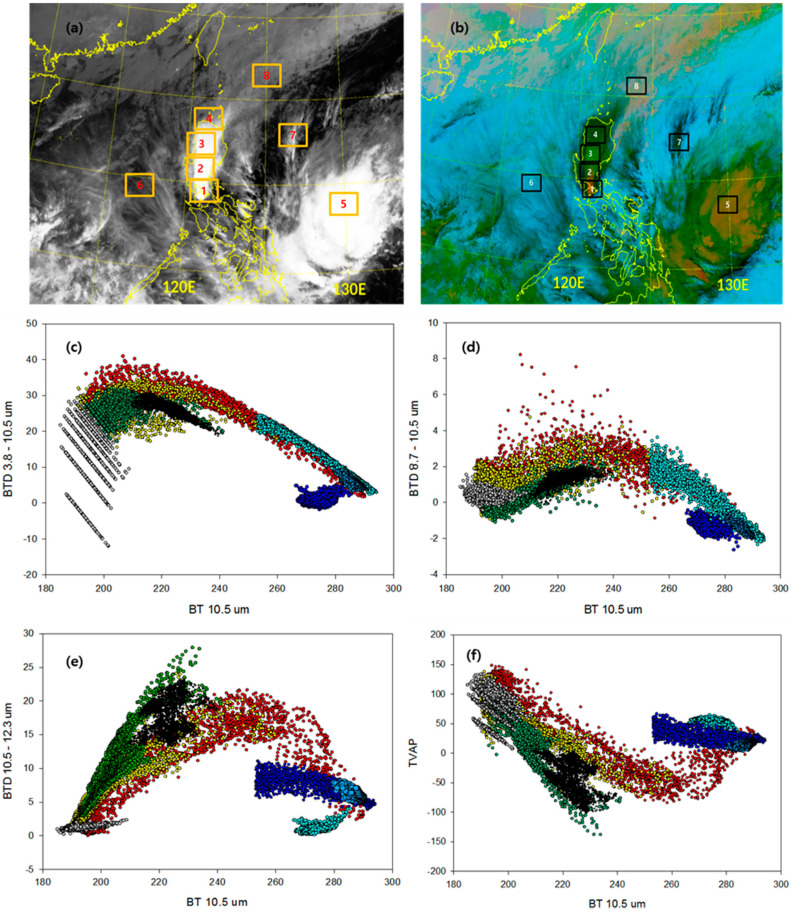
Interpretation of the Taal volcano scene divided by the color of the GK-2A Ash RGB (12 January 2020, 13:00 UTC). The images represent the GK-2A/AMI 10.5 μm brightness temperature (**a**,**b**) GK-2A Ash RGB (area 1: ash (red), area 2: mixed (yellow), area 3: SO_2_ (green), area 4: mixed (black), area 5: ice cloud (gray), area 6: clean (blue), area 7: cirrus (sky blue), area 8: water cloud (emerald)). Scatter plot between 10.5 μm and each threshold test with (c) BTD 3.8–10.5 μm, (**d**) BTD 8.7–10.5 μm, (**e**) BTD 10.5–12.3 μm and (**f**) TVAP divided by the colors of the GK-2A Ash RGB.

The eruption of the Taal volcano (14.002° N, 120.993° E) in Batangas, Philippines, on 12 January 2020, was a phreatomagmatic eruption. Ash spewed from the main crater across Calabarzon, Metro Manila, and several parts of the Central Luzon–llocos region, resulting in the suspension of school classes, work, and flights in the area. Taal is a complex volcanic system, which is hundreds of meters high and flooded by a large lake. Figure 5a shows a sequence of the IR channel at 10.5 μm from GK-2A, obtained on January 12 at 13:00 UTC, indicating that the volcano continued to spew ash as the initial plume was transported northward by prevailing winds. The Ash RGB images (Figure 5b) reveal a color difference. The plume exhibits color typical for ash (red, area 1), SO_2_ (green, area 3), or a mixture of them (yellow, area 2). An ash-contaminated water cloud can be observed, which is in the form of thin cirrus on the fringes (sky blue, area 8), a sign that the plume evolved in very humid air (lake-water steam), and the volcanic materials acted as condensation nuclei (black, area 4). The ash (red) can be easily detected in Figure 5e using the feature detection scheme, as indicated by the BTD 10.5–12.3 ranging from 0 to 20 K (“U” shape). The absorption of high concentrations of water vapor [50] can obscure the ash signal. This effect was observed in Prata in 1993 [51] and during the Taal eruption. To detect atmospheric features, such as clouds and aerosols, we applied the BTD 3.8–10.5 (Figure 5c), but we used a much higher threshold of 10 K. Figure 5b shows that ash (red), SO_2_ (green), and mixed aerosols (yellow) that have a BTD 3.8–10.5 > 17 K were detected with this test, while the water (sky blue), cirrus (pastel blue), and ocean (blue) pixels have a BTD 3.8–10.5 < 17 K. The ash is mostly associated with a BTD 3.8–10.5 that is slightly higher than 17 K, whereas both cloud types have much higher values, especially the water cloud. The BTD 3.8–10.5 test helps to prevent the misclassification of cloud-free thick aerosols as clouds because cloud-free aerosol pixels are associated with a BTD 3.8–10.5 > 17 K mostly near their source regions, such as emitted ash from the Taal volcano. The characteristics of a volcanic eruption change over time, similar to an ice cloud (gray, area 5), based on the BTD 8.7–10.5 (Figure 5d) and TVAP (Figure 5f). Therefore, false detection is possible. The patterns also show white shades typical for water (area 8) and ice clouds (area 4) at 10.5 μm at 13:00 UTC. Based on these results, two eruption stages can be identified, one at low levels and a second one that fanned out, probably below the tropopause. The second plume cast a shadow on the lower plume. Although the spatial resolution is lower, the cloud tower can be identified as a very cold spot (~192 K) in the IR band surrounded by a relatively warm ring. This cloud formation is similar to a pyrocumulus but of volcanic origin.

**Figure 6:**
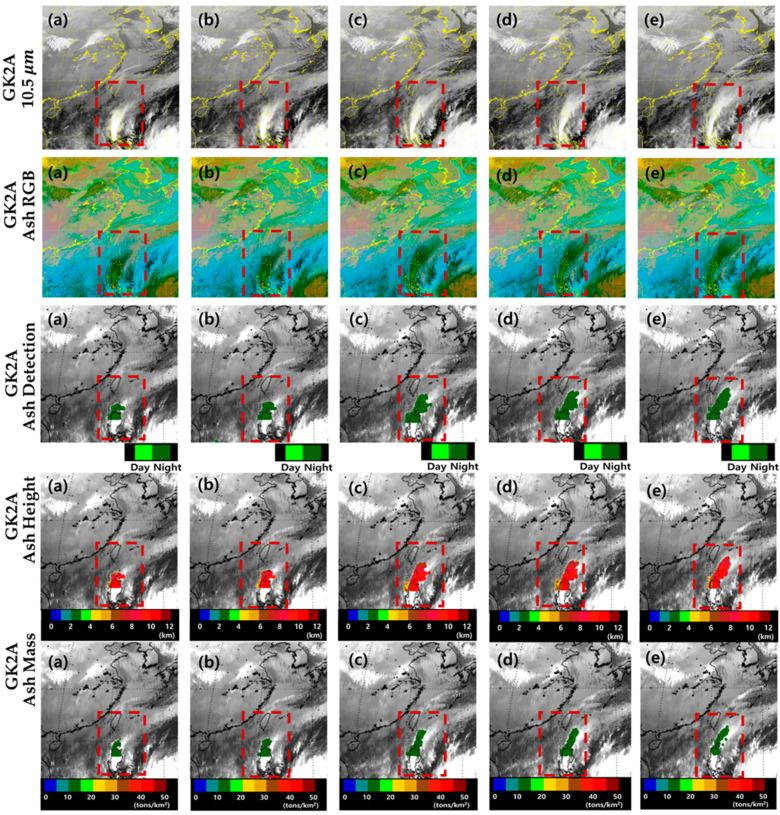
Images of the Taal volcano eruption (red dashed box) based on GK-2A BT 10.5 μm, GK-2A Ash RGB, GK-2A volcanic ash detection (VAD), GK-2A volcanic ash height (VAH), and GK-2A volcanic ash mass (VAM) on 12 January 2020 (**a**) 14:00, (**b**) 15:00, (**c**) 16:00, (**d**) 17:00, and (**e**) 18:00 UTC. The image boundary is a rectangular region (latitude 12° N–37° N and longitude 105° E–132° E; Philippines Expansion Area).

Figure 6 shows the 10.5 μm band, Ash RGB, VA detection (VAD), VA height (VAH), and VAM obtained by GK-2A on 12 January from 14:00 to 18:00 UTC at 1 h intervals based on the use of the improved algorithm. Based on the use of the previous algorithm, only the ocean could be observed during the night. However, based on the use of the improved algorithm, both land and ocean can be detected during the night, reducing the discontinuity between day and night and speeding up the detection. Over time, the number of VA pixels increases and the pixels gradually spread (night ash: dark green) from land to ocean. In addition, the VAH and concentration of VA were calculated to increase the number of VA pixels. Based on the improved algorithm, the VAH is 10–12 km (red) and the VA concentration is 10–15 tons/km^2^ (dark green), which was used as satellite data of GK-2A to obtain quantitative values as well as volcanic ash detection. This was not only used in detail for volcanic ash forecasting, but will be used as verification data for other satellites later.

#### 3.1.2. Case 2: Nishinoshima Volcanic Eruption (30 July–2 August 2020)

**Figure 7:**
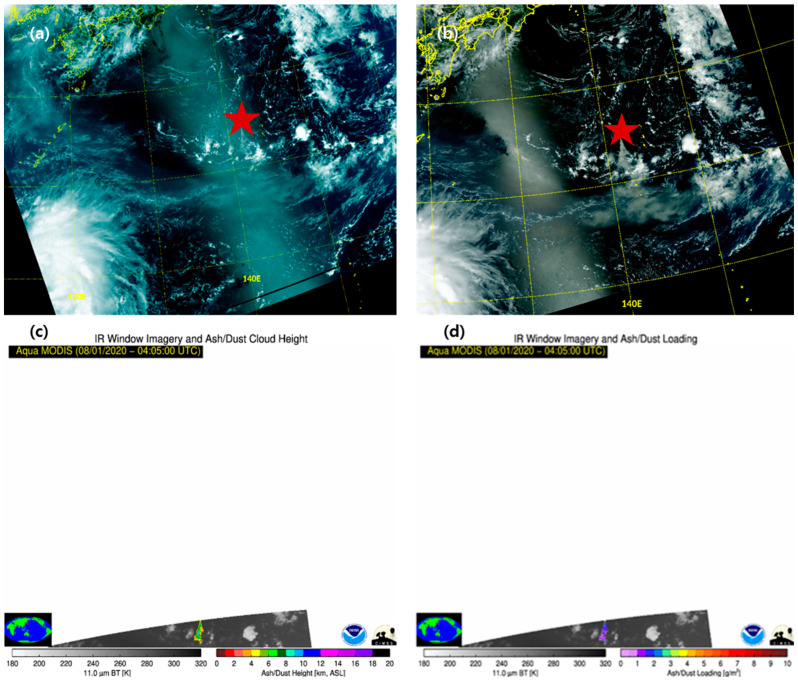
Images of low Earth orbit (LEO) satellites in the Nishinoshima area obtained on 1 August 2020. (**a**) Suomi-NPP/VIIRS True color RGB (03:38 UTC), (**b**) Aqua/MODIS (MODerate resolution Imaging Spectroradiometer) True color RGB (04:07 UTC), (**c**) Aqua/MODIS image of the ash/dust cloud height (04:05 UTC), (**d**) Aqua/MODIS image of ash/dust loading (04:05 UTC). The images can be accessed at https://volcano.ssec.wisc.edu/imagery/view/ (accessed on 10 February 2021, Space Science and Engineering Center at the University Wisconsin; NOAA (National Oceanic and Atmospheric Administration)/CIMSS (Cooperative Institute for Meteorological Satellite Studies)).

Nishinoshima is a small volcanic island (27.247° N, 140.878° E) approximately 150 km west of Chichijima and ~1000 km south–southeast of Tokyo. It has grown in size over the past decades because of volcanic activity. Several earthquakes have occurred on the island since 2020. Nishinoshima is continuously spewing ash into the atmosphere. The scales of the eruptions were small, but they lasted approximately 4 days and occurred in a very moist and cloudy environment. The second eruption was captured by Suomi-NPP/VIIRS (Suomi-National Polar-orbiting Partnership Visible Infrared Imaging Radiometer Suite) on 1 August 2020, at 03:38 UTC (Figure 7a) and Aqua MODIS (MODerate resolution Imaging Spectroradiometer) RGB on 1 August 2020, at 04:07 UTC (Figure 7b). The figures show that it spread in the shape of a triangle and VA is visible. In addition, NOAA (National Oceanic and Atmospheric Administration)/CIMSS (Cooperative Institute for Meteorological Satellite Studies) provided VAPs (height, mass) based on volcanic cloud monitoring (https://volcano.ssec.wisc.edu/) (accessed on 10 February 2021). The results recorded by Aqua MODIS on 1 August at 04:05 UTC indicate an ash/dust height of 4–7 km (Figure 7c) and ash/dust loading of 0–1.5 g/m^3^ (Figure 7d). It has been estimated that the explosiveness was lower and the VA concentration was smaller than those of general volcanic eruptions.

**Figure 8:**
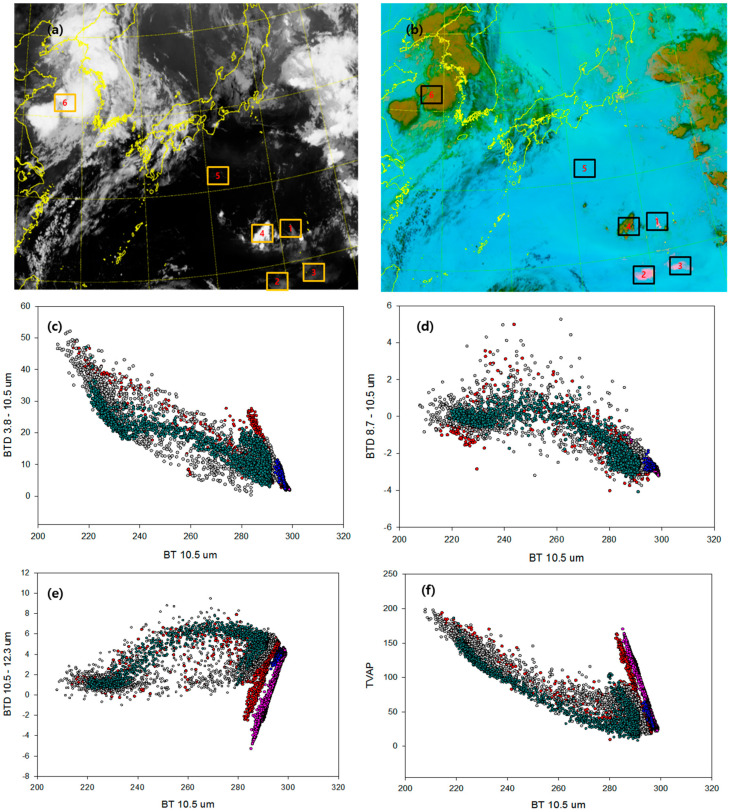
Interpretation of the scene for the Nishinoshima volcano divided by the colors of GK-2A Ash RGB (1 August 2020, 00:00 UTC). Images represent (**a**) GK-2A/AMI BT 10.5 μm and (**b**) GK-2A Ash RGB (area 1: ash (red), area 2: ash (hot pink), area 3: ash (pink), area 4: convective cloud (emerald), area 5: clean (blue), area 6: ice-cloud (gray)). Scatter plot between 10.5 μm and each threshold test using (**c**) BTD 3.8–10.5 μm, (**d**) BTD 8.7–10.5 μm, (**e**) BTD 10.5–12.3 μm and (**f**) TVAP divided by the colors of GK-2A Ash RGB.

Figure 8a,b (Ash RGB) was obtained at 10.5 μm with GK-2A at 00:00 UTC on 1 August. The Ash RGB images created using the GK-2A bands 8.6, 10.5, and 12.3 μm display an almost continuous volcanic cloud emanating from Nishinoshima during the eruption. Brighter shades of pink in the Ash RGB images suggest a higher concentration of ash within the volcanic cloud. The volcanic ash plume gradually and evenly spreads from Nishinoshima (area 1) and toward the southwest (area 2) and southeast (area 3). The more it spreads, the darker the RGB colors appear. The location of the eruption (red) in Figure 8c–e was detected by using the feature detection scheme, as indicated by the BTD 10.5–12.3 ranging from −4 to 4 K when the BT at 10.5 μm is 270–280 K. Convective clouds (gray, area 5) that formed near the volcano and cumulonimbus that formed near the Korean Peninsula (crayon, area 6) have characteristics similar to those of volcanoes and ice clouds; therefore, the distinction is ambiguous. The presence of substantial amounts of ice in the ash cloud observed in this study can thus partially or completely obscure the ash signal [7]. In addition, Potts and Ebert reported negative BTD 10.5–12.3 values over tropical areas of Asia in the absence of ash particles, but deep convective clouds and associated cold cloud tops were observed [44]. Deep convective clouds that overshoot the tropopause can cause such negative values. Because of the temperature inversion that exists at this altitude, the radiation at 10.5 μm is emitted from a cooler level than the 12.3 μm radiation [52]. However, the analysis of the BTD 10.5–12.3 and BT 10.5 μm images reveals that the negative values correspond to the opaque and semi-transparent regions of convective clouds, indicating that the cloud is formed of ice crystals rather than VA.

**Figure 9:**
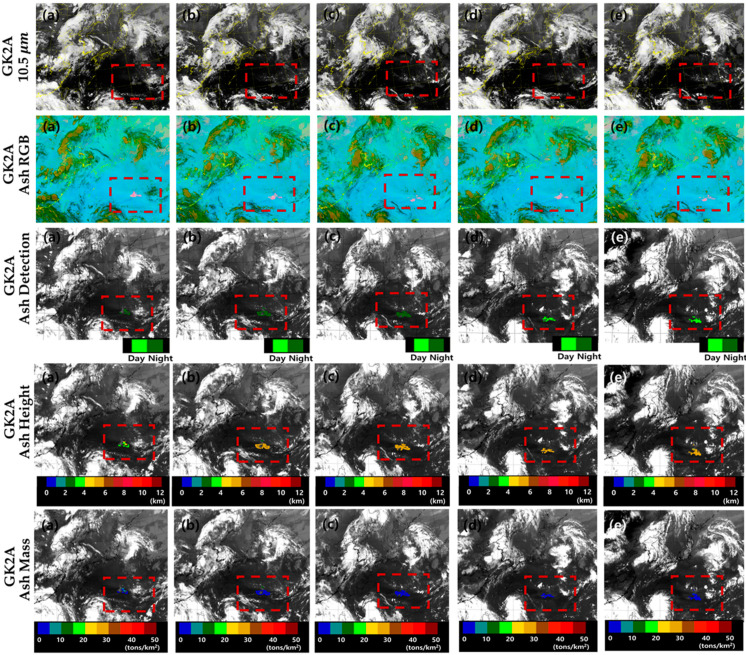
Images of the Nishinoshima volcano eruption (red dashed box) obtained on 31 July 2020, using GK-2A BT 10.5 μm, GK-2A Ash RGB, GK-2A Volcanic Ash Detection (VAD), GK-2A Volcanic Ash Height (VAH), and GK-2A Volcanic Ash Mass (VAM) at (**a**) 12:00 UTC (**b**) 16:00 UTC (**c**) 20:00 UTC and on 1 August 2020, at (**d**) 00:00 UTC (**e**) 04:00 UTC. The image boundary is a rectangular region (latitude 18° N–47° N and longitude 112° E–155° E; Japan Expansion Area).

Figure 9 shows the results obtained at 10.5 μm from 31 July, 12:00 UTC, to 1 August, 04:00 UTC, at 4-hour intervals using GK-2A and the improved algorithm (Ash RGB, VA detection, VAH, VAM). This volcanic eruption was not strong in the beginning, but it lasted for 4 days. In this study, the detection of VA pixels was poor in the beginning. However, the number of VA pixels gradually increased. This VA algorithm is detected with different thresholds for day and night, which was detected as green on 31 July, 20:00 UTC (Figure 9c), and yellow-green on 1 August, 00:00 UTC (Figure 9d). It represents the time change from night to daytime. In this case, the continuity between day and night is considered and the direction of the ash flow can be confirmed. In addition, from 12:00 UTC (Figure 9a) to 16:00 UTC (Figure 9b), the VAH increased from 4 to 6 km, but the ash mass loading remained constant. Compared with Figure 7 (Aqua/MODIS), the ash height and concentration are similar and highly accurate.

### 3.2. Validation

#### 3.2.1. Case 1: Taal Volcano Eruption (12 January 2020)

**Figure 10:**
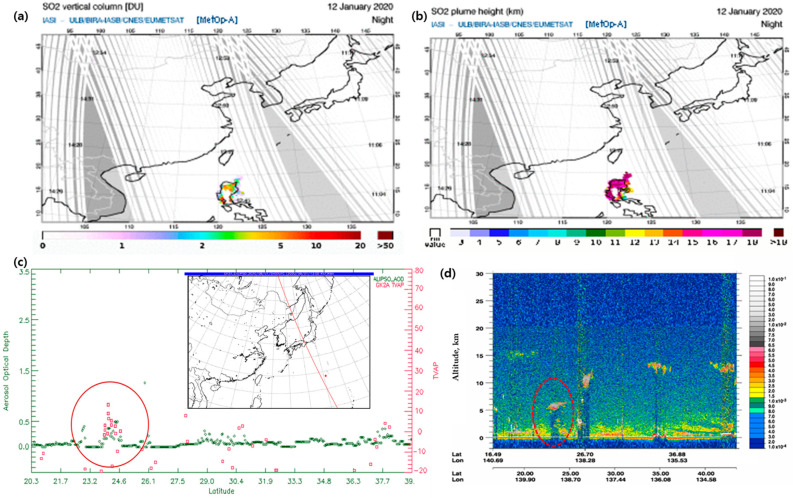
Images of MetOp-A/IASI for the Taal volcano eruption on 12 January 2020 (**a** and **b**), and Cloud-Aerosol Lidar and Infrared Pathfinder Satellite (CALIPSO) for the Nishinoshima volcano eruption at 04:06 UTC on 1 August 2020 (**c** and d). (**a**) SO_2_ vertical column and (**b**) SO_2_ plume height of MetOp-A/IASI provided by the Aviation Control Service (SACC: https://sacs.aeronomie.be/nrt/) (accessed on 10 February 2021). (**c**) Analysis of the AOD of CALIPSO (Column Optical Depth Aerosols 532 nm) and TVAP of GK-2A after collocations when the CALIPSO satellite passed through the Nishinoshima region (marked by the red circle) and (**d**) 532 nm total attenuated backscattering (marked by the red dotted circle) from CALIPSO.

Figure 10a,b shows the SO_2_ detected using MetOp-A (Meteorological Operational satellite program of Europe-A)/IASI; (Infrared Atmospheric Sounding Interferometer) during nighttime on 12 January 2020, following the start of the eruption. The IASI data provide the SO_2_ in the presence of ash as well as coverage over polar regions beyond the SEVIRI (Spinning Enhanced Visible and Infra-Red Imager) field [53]. A large portion of the SO_2_ was spread over the Taal crater in the Philippines into the northeast. The vertical SO_2_ detected by IASI was 3–10 DU (Dobson Unit). The height of the IASI SO_2_ plume was calculated to range from 12 to 19 km. The VAH based on GK-2A was calculated to be 12 km. However, the amount of volcanic ash cannot be verified because the data are insufficient.

#### 3.2.2. Case 2: Nishinoshima Volcano Eruption (31 July–2 August 2020)

The CALIPSO provides a wealth of actively sensed data over the region and thus an outstanding research opportunity [54]. We used CALIPSO data to compare the vertical distribution of VA and AOD during the volcanic eruption. Because CALIPSO is an active sensor, it can only be used when passing a place during a volcanic eruption. The CALIPSO satellite passed the Nishinoshima eruption site in Japan at 04:06 UTC on 1 August 2020 (Figure 10c). Based on the comparison of the TVAP obtained by GK-2A and the AOD (Column_Optical_Depth_Aerosols_532) measured by CALIPSO (CAL_LID_L2_05kmAPro-V3-40.2020-08-01T04-06-05ZD, https://search.earthdata.nasa.gov/) (accessed on 10 February 2021), which are representative indices for the detection of VA, the TVAP and AOD simultaneously increased 4 km from Nishinoshima. The AOD obtained by CALIPSO was low in the range of 0 to 0.5, but it showed a tendency to increase when compared to other regions. The TVAP based on GK-2A was within the threshold of the GK-2A VA detection used in this study; it ranged from −20 to 20 K. The CALIOP (Could-Aerosol Lidar with Orthogonal Polarization) is designed to acquire vertical profiles of elastic backscattering at a wavelength of 532 nm using a near-nadir-viewing geometry during both day and night phases of the orbit. These profiles can be used to determine the presence of clouds and aerosols, where a higher backscattering value indicates a higher aerosol concentration (it also depends on the shape and radius of the aerosols). Based on the analysis of the profiles, strong backscattering (532 nm) was observed at the point coinciding with the previous collocation spot (i.e., higher concentrations of aerosols), indicating an altitude of 5 to 6.5 km at the same point as the GK-2A VAH (Figure 10d). Although the VAH based on GK-2A is somewhat underestimated compared to CALIOP data, it is meaningful that it was used for volcanic forecasting using GK2A.

### 3.3. Limitations of GK2A/AMI VAP Algorithms

This study has a possible dependence of the chosen threshold on the environment and observation conditions of six cases of volcanoes in 2020. So, it is a threshold value limitedly determined for a specific case of a volcanic eruption, and it is used as a background threshold value using a 30-day background field before volcanic ash detection. This is a threshold value reflecting seasonal fluctuations and characteristics that exit when the volcano erupted, so there is a limit. Additionally, in the case of performing the validation of the Taal volcano on 12 January it was used as a qualitative analysis of SO_2_ height (km) related to the volcano. This is because direct comparison was difficult due to the lack of a ground truth (actual measurement), and although it is SO_2_ related to volcanic eruption, there is a limit to the validation dataset because of difference paths between ash and SO_2_ plumes, the algorithm, characteristics between volcanic ash and SO_2_ emissions.

## 4. Conclusions

Based on the improved VAP algorithm of GK-2A, multispectral data could be obtained for volcanic plumes. To compensate for the VA effect, various threshold tests have been developed for ash detection. The clustering algorithm makes use of GK-2A channels that are known to be useful for the detection of VA (0.6 and 3.8 μm reflectance and 10.5 μm). In addition, several channels, that is, BTD 3.8–10.5, BTD 8.7–10.5, BTD 10.5–12.3, and BBTD, were selected based on PCA. A three-band combination (GK-2A Ash RGB, TVAP) was utilized to determine the thresholds for six volcanic eruptions that occurred in 2020. A new threshold, that is, BTD 8.7–12.3, was introduced, which is based on the absorption of SO_2_ and the utilization of various 3.8 μm channels, to increase the accuracy at night. The algorithm was changed to facilitate the detection of both land and ocean at night. The discontinuity between day and night was reduced by adjusting the boundary values between daytime and nighttime. Based on the improved VAP algorithm using GK2A/AMI, the Sinabung eruption in Indonesia that occurred on 10 August 2020, could be clearly identified. The results of this study indicate that this VAP can be used to prevent damage due to future volcanic activity and to yield a higher detection accuracy. The advantage of this VAP of GK2A/AMI is that it not only uses a lot of the existing boundary values detected by volcanoes, but also tries to increase the accuracy by using an additional 3.8 μm. In particular, by easing the application method of cloud detection, it is possible to distinguish between volcanic ash clouds and cumulus, convective initiation. Therefore, it is improved to detect both strong and weak volcanic eruptions. It is encouraging that after launching the satellite of GK2A/AMI, the sensor is used for the first volcanic eruption detection, and quantitative volcanic ash amount and altitude are estimated and used for forecasting. However, since the number of volcanic cases is limited to six, the threshold is unstable, and there is a need for continuous tuning. Through improvement activities such as tuning work in the future, its use in association with high-quality weather observations and accurate weather forecasts is particularly important for the safe operation of aircrafts.

## Figures and Tables

**Table 1:** Summary of the GEOKOMPSAT-2A (GK-2A)/Advanced Meteorological Imager (AMI) spectral bands.

Bands	Band Name	Wavelength		Band Width (Max)	Spatial Resolution (km)
		Min (μm)	Max (μm)		
1 (blue)	VIS0.47	0.43	0.48	0.075	1
2 (green)	VIS0.51	0.52	0.52	0.063	1
3 (red)	VIS0.64	0.63	0.66	0.125	0.5
4 (VIS)	VIS0.86	0.85	0.87	0.088	1
5 (NIR)	NIR1.37	1.37	1.38	0.030	2
6 (NIR)	NIR1.6	1.60	1.62	0.075	2
7 (IR)	SWIR3.8	3.74	3.96	0.500	2
8 (IR)	WV6.3	6.06	6.43	1.038	2
9 (IR)	WV6.9	6.89	7.01	0.500	2
10 (IR)	WV7.3	7.26	7.43	0.688	2
11 (IR)	IR8.7	8.44	8.76	0.500	2
12 (IR)	IR9.6	9.54	9.72	0.475	2
13 (IR)	IR10.5	10.3	10.6	0.875	2
14 (IR)	IR11.2	11.1	11.3	1.000	2
15 (IR)	IR12.3	12.2	12.5	1.250	2
16 (IR)	IR13.3	13.2	13.4	0.750	2

**Table 2:** List of the input variables used for the calculation of the volcanic ash aerosol optical depth (AOD) lookup table.

Variable Name	Number of Entries	Entries
Wavelength	5	3.8, 10.5, 11.2, 12.4, 13.3 μm (considering spectral response function)
Solar zenith angle	9	0, 10, 20, 30, …, 80 (10 intervals)
Satellite zenith angle	17	0, 5, 10, 15, …, 80 (5 intervals)
Relative azimuth angle	18	0, 10, 20, …, 170 (10 intervals)
AOD	10	0.0, 0.3, 0.6, 0.9, 1.2, 1.5, 2.0, 3.0, 4.0, 5.0
Volcanic ash model	10	WCP-112, 1986 [32] (considering effective radius 1, 2, 3, 4, 5, 6, 7, 8, 9, 10 μm)
Altitude	10	1, 2, 3, 4, 5, 6, 7, 8, 9, 10 km

**Table 3:** Volcanic eruptions in 2020.

Date, 2020.	Location	Latitude	Longitude	Analysis Time
12 January	Taal, Philippines	14.00	120.99	16:00 UTC, 12 January 2020
2 March	Merapi, Indonesia	−7.54	110.45	23:00 UTC, 2 March 2020
27 March	Merapi, Indonesia	−7.54	110.45	06:00 UTC, 27 March 2020
11 April	Krakatau, Indonesia	−6.10	105.42	18:00 UTC, 10 April 2020
16 May	Smeru, Indonesia	−8.11	112.92	12:00 UTC, 16 May 2020
30 July–4 August	Nishinoshima, Japan	27.24	140.87	16:00 UTC, 31 July 2020

## Data Availability

Data available on request due to restrictions eg privacy or ethical. The data presented in this study are available on request from the corresponding author.
